# “How Come You Don’t Call Me?” Smartphone Communication App Usage as an Indicator of Loneliness and Social Well-Being across the Adult Lifespan during the COVID-19 Pandemic

**DOI:** 10.3390/ijerph18126212

**Published:** 2021-06-08

**Authors:** Britta Wetzel, Rüdiger Pryss, Harald Baumeister, Johanna-Sophie Edler, Ana Sofia Oliveira Gonçalves, Caroline Cohrdes

**Affiliations:** 1 Mental Health Research Unit, Department of Epidemiology and Health Monitoring, Robert Koch Institute, 12101 Berlin, Germany; brittawetzel@mailbox.org (B.W.); EdlerJ@rki.de (J.-S.E.); 2 Institute of Clinical Epidemiology and Biometry, Julius-Maximilians-University of Würzburg, 97080 Würzburg, Germany; ruediger.pryss@uni-wuerzburg.de; 3 Department of Clinical Psychology and Psychotherapy, Ulm University, 89081 Ulm, Germany; harald.baumeister@uni-ulm.de; 4 Institute of Public Health, Charité—Universitätsmedizin Berlin, 10117 Berlin, Germany; ana.goncalves@charite.de

**Keywords:** loneliness, social well-being, passive data, app, smartphone communication, COVID-19, social media use, age differences, public mental health, mental health monitoring

## Abstract

Loneliness and lack of social well-being are associated with adverse health outcomes and have increased during the COVID-19 pandemic. Smartphone communication data have been suggested to help monitor loneliness, but this requires further evidence. We investigated the informative value of smartphone communication app data for predicting subjective loneliness and social well-being in a sample of 364 participants ranging from 18 to 78 years of age (52.2% female; mean age = 42.54, SD = 13.22) derived from the CORONA HEALTH APP study from July to December 2020 in Germany. The participants experienced relatively high levels of loneliness and low social well-being during the time period characterized by the COVID-19 pandemic. Apart from positive associations with phone call use times, smartphone communication app use was associated with social well-being and loneliness only when considering the age of participants. Younger participants with higher use times tended to report less social well-being and higher loneliness, while the opposite association was found for older adults. Thus, the informative value of smartphone communication use time was rather small and became evident only in consideration of age. The results highlight the need for further investigations and the need to address several limitations in order to draw conclusions at the population level.

## 1. Introduction

Loneliness is associated with an increased mental and physical health burden and premature mortality [1,2,3,4,5] and has been described as a major public health concern [6]. During the COVID-19 pandemic, many countries have introduced contact restriction measures to minimize the spread of the SARS-CoV-2 virus. Given the persistent duration of these measures, questions about social well-being and loneliness have been increasingly raised. Indeed, initial reports support an elevated risk of loneliness. For example, a German survey conducted from April to June 2020 investigated the socioeconomic consequences of the SARS-CoV-2 virus (the SOEP-CoV-Study) and found that loneliness increased in the adult population during the early phases of the pandemic in Germany [7]. In line with these findings, a nationally representative study from the UK showed that 35% of the participants reported feelings of loneliness during the pandemic [8]; similarly, a report from the US found a level of loneliness that was 43% above previously identified levels [9].

There are several ways to measure loneliness on a populational level, including self-reported interviews and questionnaires [10]. Such retrospective and self-reflective methods rely on memories and competencies and may be influenced by reporting or recall bias [10]. With the availability of digitalization and new data collection opportunities, research on the use of passive smartphone data to investigate mental health outcomes such as loneliness has increased [11,12,13,14,15]. The early and reliable detection of loneliness is important to initiate targeted interventions for specific risk groups [16]. Thus, it has been suggested that passive smartphone data could be used in (public) mental research to overcome the limitations of other data collection methods [11]. One potential benefit is that passive smartphone data are continuously available and allow the investigation of trends over time [11,17,18]. Moreover, a variety of data, such as location visits, sleep time and phone use time, can be monitored [11]. Additionally, smartphones are easy to use, cheaper than other measurement devices, and are often carried throughout the day [19,20]. Another advantage is that interactive features, such as feedback or recommendations, can be included in passive monitoring systems [20,21]. However, there are also some risks and challenges that should be considered [15]. For example, although the use of smartphones has grown in high-income countries [22], certain population groups, such as older people, are still underrepresented in passive smartphone sensing studies (the so-called usage gap) [23]. In addition, people may not consent to have their mental health status monitored, and fear of stigma can also be an obstacle [24,25]. Furthermore, privacy and technological concerns, as well as sensor precision, require further progress and investigation [17]. For example, the validity of using passive smartphone data to monitor mental health outside of the clinical context is still limited [26]. During the COVID-19 pandemic, when face-to-face contacts and clinical visits are limited, the use of smart and/or digital health devices has gained importance to prevent or reduce the mental health burden in the general population [16,27].

### 1.1. Loneliness and Social Well-Being

Whereas social isolation can be defined as an objective separation from social contacts [5,28], loneliness refers to the perceived discrepancy between desired and actual social contacts [29]. As suggested by Keyes [30], feeling socially connected represents one central component of social well-being, in addition to social actualization, social coherence, social integration, social contribution, and social acceptance. Indeed, a feeling of social connectedness represents an essential part of individual health according to the World Health Organization [31]. Feeling lonely and not socially connected increases the risk of coronary heart disease, increased blood pressure, and stroke [2,3,4] and is associated with lower sleep quality, depressive symptoms and suicidal ideation [5,32,33,34]. A lack of perceived social connection has also been related to health behaviors. People who feel lonely are more likely to have reduced medical compliance, increased smoking and alcohol use, less physical activity, and a greater likelihood of being obese than people who do not feel lonely [2,35,36,37]. Hence, interventions aiming to reduce loneliness are considered crucial to minimize the suffering of individuals and to decrease health care costs [38]. Feelings of loneliness and social well-being have often been treated separately, and although theoretically inversely related, simultaneous consideration is relatively rare, particularly in connection with the COVID-19 pandemic.

Studies that have focused on how loneliness varies over the adult life span suggest a u-shaped association between age and loneliness: young (less than 30 years) and very old people (older than 85 years) report feeling lonely, while middle-aged people report lower levels of loneliness [39,40,41]. These trends have also been observed during the COVID-19 pandemic. For instance, the German SOEP-CoV study showed that young adults in particular reported feeling lonely during the early phases of the COVID-19 pandemic due to contact restrictions [7]. The finding that younger people felt lonelier than older people during the COVID-19 pandemic has been replicated in several other studies [8,42,43]. In addition, concerns about increasing loneliness among older adults have been raised during the COVID-19 pandemic, particularly for those living in retirement homes [16]. Ways to engage older people in social activities, such as volunteer work, are restricted. Thus, older people often spend most of their time alone [16]. Due to the smartphone usage gap, which refers to the unequal usage distribution among different age, sex, or income groups, older people are less likely than younger people to stay in contact with their social networks via smartphones or tablets [23]. Apart from age, sex, education, and family status have also been proposed as risk factors for loneliness. Studies suggest that women feel lonelier than men [7,41] and that people with a low education level, low income, or those who live alone experience increased loneliness [44].

### 1.2. Associations with Passive Smartphone Communication Data

The various types of smartphone communication function, such as phone calls or social media apps, offer the opportunity to connect with others [45]. Previous studies have revealed associations between loneliness and social well-being and the frequent use of smartphone communication functions, such as social media, instant messenger and video call apps, phone calls, and short text messages [18,45,46,47,48,49]. The results of the longitudinal study of Lapierre et al. [50] underline the impact of problematic smartphone use on well-being by showing that smartphone addiction predicts later loneliness and depressive symptoms.

#### 1.2.1. Social Media Apps

The relationship between social media use and loneliness remains unclear. If social media is used to maintain existing relationships, it seems to reduce loneliness [51]. However, social media apps may actually increase loneliness when they are used to replace real-world contacts loneliness [51]. According to Nowland et al. [51], age seems to be an important moderator of the association between loneliness and social media use. Song et al. [52] suggest that further studies should focus on age differences because the association between Facebook use and loneliness may differ across the adult age range. However, many studies on social media use and loneliness have been conducted with young participants (e.g., college students) only, and the findings are inconsistent [45]. For instance, the interactive and passive use (browsing) of Instagram among college students was associated with lower levels of loneliness, while Instagram broadcasting was related to higher levels of loneliness [53]. Among young people (18–35 years), the use of Facebook was associated with social support and psychological well-being, while the number of Facebook friends was associated with negative feelings (e.g., feeling angry or afraid) and “entrapment”, which is the perception of stress due to the pressure of having to be available to others [45]. However, the longitudinal data of Dissing et al. [54] emphasized the relevance of analyzing course data. While in their baseline survey, high levels of smartphone use were associated with lower levels of loneliness, in the follow-up survey, a large number of Facebook contacts and a long duration of phone calls (for women) were associated with increased loneliness [54]. Primack et al. [47] found that among young adults (19–32 years), a short duration of social media use (0 to 29 min per day) was associated with lower levels of loneliness, while a longer duration (greater than 120 min per day) was related to increased loneliness. The evidence from studies that included older age groups seems to be more consistent—the use of social media and technology is related to lower levels of perceived loneliness and higher levels of feeling socially connected among older adults [46,51,55,56,57].

#### 1.2.2. Instant Messenger Apps

Similar to social media apps, differences have been observed between age groups in their use of instant messenger apps. In a study by Chan [45], the use of WhatsApp was related to perceived social support in the youngest age group (18–35), while the number of WhatsApp groups was associated with feelings of entrapment. In the same study, a positive relationship between WhatsApp use and psychological well-being was found only in the oldest age group (55 to 70+ years) [45]. In line with these findings, a study by Chopik [46] found that older adults (50+ years) had positive attitudes toward social technology (e.g., instant messaging, social media, or e-mail). The author concluded that social technology may contribute to stronger and satisfactory relationships among older adults [46].

#### 1.2.3. Phone Calls and Short Text Messages

Chan and Li [58] found that mobile voice communication directly predicted subjective well-being. The number of sent and received short text messages was associated with decreased levels of loneliness due to higher levels of relationship satisfaction [59]. An app-based mobile sensing study found that lonely individuals received fewer incoming phone calls and short text messages than individuals who did not report feeling lonely [18].

#### 1.2.4. Video Calls

Previous studies have investigated the association between loneliness and the use of video calls in older adults in care settings [48,60]. A review by Noone et al. [61] showed that the use of video calls had little or no effect on loneliness. However, according to the authors, the studies were conducted with small numbers of participants, and the methodologies used require improvement [61].

### 1.3. The Present Study

Many studies on passive smartphone communication and subjective loneliness or social well-being have used restricted sample populations (e.g., college students) and have often focused on one specific type of smartphone communication [45]. However, the validation of passive monitoring requires testing based on diverse population groups and smartphone communication types over an adequate period of time to generate robust conclusions [20]. It has been pointed out that research should focus on the validity and acceptance of using passive smartphone data to monitor mental health [17,26]. Therefore, the current study investigated the associations between the diverse smartphone communication data and self-reported loneliness and social well-being. We investigated the extent to which smartphone communication app use can add informative value for monitoring loneliness and social well-being in the general population. In addition, we analyzed age differences in the associations between passive smartphone communication data and self-reported loneliness and social well-being. We also considered differences in the associations among the different types of communication.

Based on previous evidence and theoretical assumptions, we expected that smartphone communication app use is associated with self-reported loneliness and social well-being (Hypothesis 1; H1). In addition, we expected the associations to differ among the diverse types of communication apps. More precisely, we expected to find a positive association between frequent phone calls and short text messages and social well-being, and a negative association with self-reported loneliness (H2a). Moreover, we expected to find age-related differences in the associations between social media and messenger app use and self-reported loneliness and social well-being. We believed that the strength of the positive associations would increase in the older age groups (H2b). Due to previously inconclusive or missing evidence, we also explored the associations between other communication app types and self-reported loneliness and social well-being (across different ages).

## 2. Materials and Methods

### 2.1. Sample and Procedure

The CORONA HEALTH APP study is a cooperative research project of the Robert Koch Institute and the Universities of Würzburg, Ulm and Regensburg (see [62] for a detailed description). It is an ongoing study combining a cross-sectional and a longitudinal design, with weekly follow-ups that started in July 2020. Participation was voluntary, and each participant provided informed consent. The participants do not receive any financial reward but are offered news relating to the COVID-19 pandemic in a ticker, general information about counseling services, and personal feedback about their well-being (based on standardized cutoff scores for symptoms of loneliness and depression) immediately after they complete the initial questionnaire. Study participation can be canceled at any time during the questionnaire without transferring data. The questionnaire collects demographic and psychosocial health information, including subjective perceptions of loneliness and social well-being. Additionally, participants are asked to approve or reject the transfer of a selection of 10 common passive smartphone communication app use times for each day of the past week. Due to the data protection regulations of iOS devices, passive smartphone communication data can be obtained from Android smartphones only. Apple does generally not allow the gathering of passive smartphone communication data such as a user’s app usage history.

**Figure 1:**
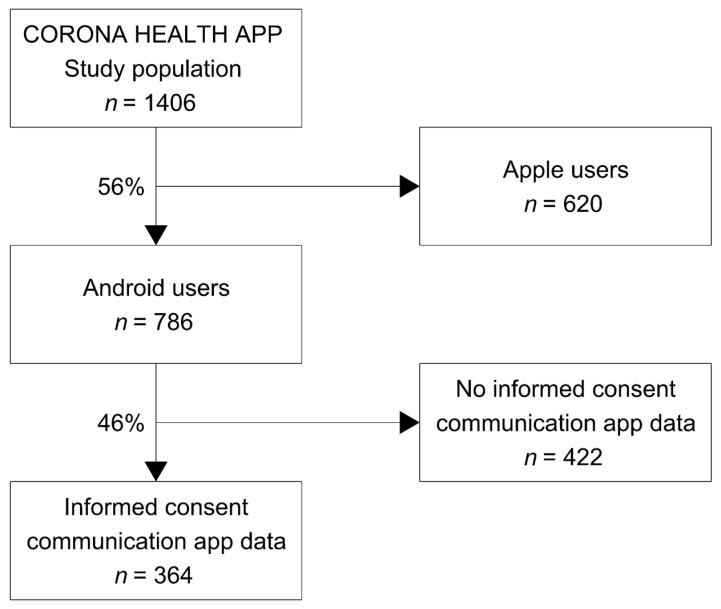
Flowchart of the current study’s sample composition.

The present analyses are based on the cross-sectional questionnaire and communication app data collected between July and December 2020. As displayed in Figure 1, the final sample size comprised 364 adults, ranging from 18 to 78 years of age (52.5% female, 46.2% male and 1.4% diverse; mean age = 42.54 years, *SD* = 13.22 years). Among the total sample of 1406 people, the proportion of Android users was 55.9%, and 46.3% agreed to transfer smartphone communication app data. Additional sample characteristics are presented in Table 1.

A priori sample size calculation suggested that a minimal sample size of *n* = 247 is sufficient for detecting a small to moderate effect of *f*^2^ = 0.10 in a multiple linear regression fixed effects model (*R*^2^ deviations from zero) with 18 predictors at a power level of 0.90 and with an error probability of 0.05.

### 2.2. Measures

#### 2.2.1. Loneliness

We measured self-reported loneliness with the Loneliness Scale-SOEP [LS-S] [63], which includes the following three items: “How often do you feel that you lack companionship?”, “How often do you feel left out?”, and “How often do you feel isolated from others?”. Questions were answered from 1 (very often) to 5 (never). We inversely recoded the items according to the coding scheme and summed the items to a total score, with higher values indicating higher self-reported loneliness, ranging from 3 (min) to 15 (max). The internal consistency was good, with a Cronbach’s alpha of 0.81. The studies of Buecker et al. [64] and Beutel et al. [65] are examples of the application of the Loneliness Scale-SOEP.

#### 2.2.2. Social Well-Being

We measured social well-being with a subscale of the World Health Organization Quality of Life-Instrument (WHOQOL-Bref) [66], which includes the following three items: “How satisfied are you with your personal relationships?”, “How satisfied are you with your sex life?”, and “How satisfied are you with the support you get from your friends?”. Answers varied from 1 (very dissatisfied) to 5 (very satisfied). As described in the WHOQOL user manual, we transformed the answers into an overall score ranging from 1 (min) to 100 (max). Higher values indicate better social well-being [62]. The internal consistency was acceptable, with a Cronbach’s alpha of 0.73. The WHOQOL-Bref has been used in several international studies within the context of the COVID-19 pandemic such as in the study of Algahtani et al. [67] as well as within the context of smartphone use as in Li et al. [68].

#### 2.2.3. Passive Smartphone Communication App Data

We collected smartphone communication app use times for each day of the last 7 days prior to answering the questionnaire for a selection of 10 smartphone communication apps (Facebook, Facebook Messenger, Instagram, Snapchat, WhatsApp, Telegram, Skype, Skype Messenger, Zoom, phone calls, and SMS). “Use time” refers to the time an app was actively used by an individual and not, for instance, passively open in the background. Time was measured in milliseconds per day. We first transformed milliseconds into minutes and seconds per day and averaged the daily use times across the week. In a second step, we assigned all apps into five smartphone communication categories in accordance with previous research [49,69,70,71,72,73,74] and in consideration of discussions about the major purposes of each communication mechanism (e.g., Snapchat [71,75]). As a result, we summarized the use times of Facebook, Instagram, and Snapchat as social media; WhatsApp, Telegram, Facebook Messenger, and Skype Messenger as instant messengers; and Skype and Zoom as video calls. Phone calls and short message services were not summarized; they each constituted their own category.

#### 2.2.4. Control Variables

Based on previous indications, we included the participant’s age, sex, education status, partnership status, chronic condition, and lifetime diagnosis of a mental disorder as control variables. We measured the education status by asking participants about their highest educational degree. Next, the answers were categorized in line with the Compara-tive Analyses of Social Mobility in Industrial Nations (CASMIN) classification into three categories (low, moderate, and high) [76]. The partnership status differentiated between participants with a partner (including being married, having a permanent relationship, or being in a registered partnership for same sex couples) and participants without a partner (including being married but living apart, widowed, divorced, or unmarried).

Participants were asked whether they had ever received a diagnosis of a mental disorder (such as depression, anxiety disorder, or addiction) from a physician or psychotherapist (yes/no) or suffered from a chronic condition based on one question from the Minimum European Health Module (MEHM): “Do you suffer from any chronic or long-term illness or condition (health problem)?” (yes/no) [77]. Moreover, we asked participants to indicate whether they were currently infected with COVID-19 (yes/no).

### 2.3. Statistical Analyses

All analyses were carried out with R statistics (R Foundation for Statistical Computing, Vienna, Austria) [78]. Data inspection revealed that the assumption of normality was violated for the outcome measures (loneliness was slightly skewed to the right and social well-being was skewed to the left). Thus, we used robust standard errors for the model calculations. In addition, loess smoothing of single correlations with the outcome measures indicated a nonlinear association with the age of participants; therefore, we also included the quadratic age term in our analyses.

In line with the rule of thumb derived from simulation studies of a minimum of 10 events per variable [79], we had to exclude two small groups from the regression analyses: people of diverse sexes (*n* = 5) and video call users (*n* = 19; interactions with age groups *n* < 10). Additionally, the interaction terms of phone calls were excluded due to the small numbers of observations in each group. Thus, the final sample size for inferential statistical analyses and hypothesis testing comprised 359 female and male participants.

We used four multiple robust regression models to analyze the associations between smartphone communication app data and self-reported feelings of loneliness and social well-being. We also analyzed differences in the associations by communication app type, age, and age quadratic, which were in accordance with the research questions. In the first model (M1), self-reported loneliness was the outcome. The weekly average total use time, age, age quadratic, and the respective interaction terms of age and age quadratic with the total use time were predictors, while demographic characteristics were control variables (sex, education status, partnership status, self-reported clinician-diagnosed mental disorders, chronic conditions, and a COVID-19 infection). The second model (M2) was built similar to the first but used social well-being as the outcome variable.

In the third and fourth models, we used loneliness (M3) and social well-being (M4) as the outcome variables. The weekly averaged use times of the different types of smartphone communication apps (social media, instant messenger, phone calls, and text messages), age, age quadratic, and the interactions between use times and age and age quadratic were predictors, and the aforementioned demographic characteristics were control variables. Thereafter, we probed the significant interactions with simple slope analyses.

To investigate the additional value of smartphone communication app use in explaining variance in feelings of loneliness and social well-being, we computed each model without the use time predictors and interactions (M1.0 to M4.0) and, in a second step, we compared the goodness of fit between models (e.g., M1 vs. M1.0) by means of a likelihood-ratio (LR) test.

## 3. Results

### 3.1. Sample Description

The sample included 52.5% females, 46.2% males, and 1.4% diverse participants (Table 1). Participants were aged between 18 and 78 years (*M* = 42.57, *SD* = 13.17 years), with the majority of participants ranging in age between 30 and 59 years (70.3%). A relatively high proportion of participants had a high educational level (67.0%) and reported a lifetime diagnosis of mental or chronic disorder (43.8%; Table 1).

The total average communication app use time was 367.73 min per week and 45.965 min per day (Table 2). On average, the participants spent most of the time with social media (245.83 min per week) and instant messaging (205.64 min per week) (Table 2). Regarding general use (independent of time), instant messenger apps were used by nearly all participants (946.81%), social media by two-thirds (63.24.1%), SMS by slightly more than half (57.19%), and usual phone calls by one-third (34.95.4%). A minority of participants used video call apps (5.23%; Table 2).

### 3.2. Hypotheses Testing

**Figure 2:**
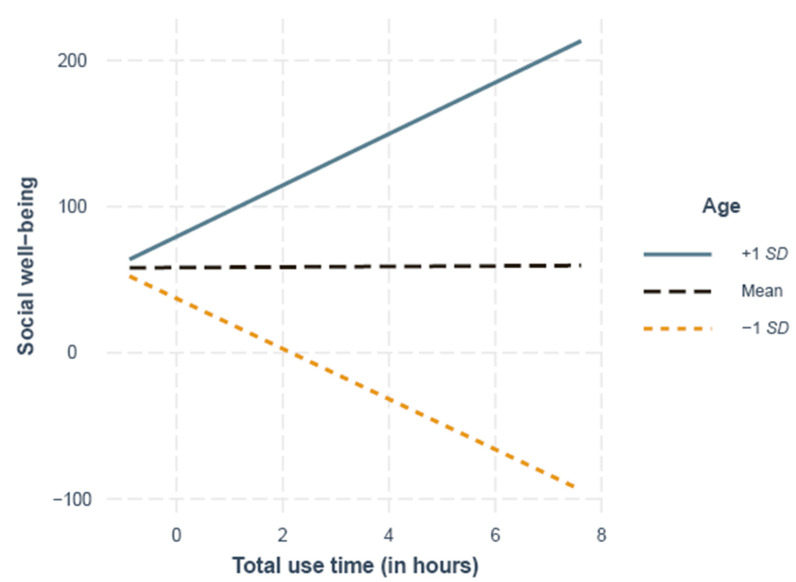
Simple slopes of the total use time and social well-being at 1 *SD* above (55 years), 1 *SD* below (29 years) and the mean age (42 years).

As presented in Table 3, the total use time was unrelated to loneliness (M1) but significantly related to social well-being (M2) when taking the age of participants into account, as indicated by the significant age as well as age quadratic and total use time interactions. The simple slope analysis results showed a significant difference from zero for the slope 1 *SD* below the mean (29 years), indicating that younger participants with a higher total use time had lower levels of social well-being (*B* = −17.22, *SE* = 8.25, *p* = 0.03; Figure 2), whereas the opposite was the case for older participants, as indicated by a significant difference from zero for the slopes at 1 *SD* above the mean (55 years; *B* = 17.59, *SE* = 8.73, *p* = 0.04). The slope at the mean age (42 years) was not significantly different from zero (*B* = 0.19, *SE* = 1.16, *p* = 0.87; Figure 2).

**Figure 3:**
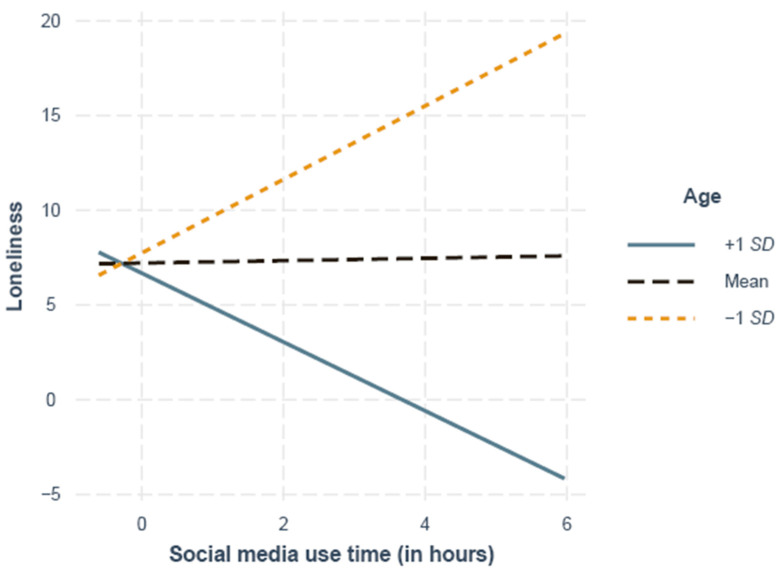
Simple slopes of the social media use time and loneliness at 1 *SD* above (55 years), 1 *SD* below (29 years), and the mean age (42 years).

M3 and M4 revealed that the instant messenger and SMS use times were not significantly related to loneliness or social well-being, but phone call use time was positively related to social well-being. There was a significant interaction of age quadratic and social media use time, indicating age-related differences in the association between social media use and perceived loneliness. The simple slope analysis showed a significant difference from zero for the slopes at 1 *SD* above the mean (55 years), indicating that older participants with a higher social media use time had significantly lower levels of loneliness (*B* = −0.29, *SE* = 0.15, *p* = 0.047). A marginally significant difference from zero for the slopes at 1 *SD* below the mean (29 years; *B* = 0.30, *SE* = 0.16, *p* = 0.057) indicated the trend that younger age and a higher social media use time were associated with higher levels of perceived loneliness. The slope of participants at the mean age did not differ significantly from zero (42 years; *B* = 0.01, *SE* = 0.02, *p* = 0.78; Figure 3).

Additionally, in all four models, we identified several associations between individual and social factors that were entered as control variables and perceived loneliness and social well-being. Consistently, the linear and quadratic terms of age were significantly related to social well-being but not to loneliness (Table 3 and Table 4). Participants with a self-reported clinician-diagnosed mental disorder reported significantly higher levels of loneliness and lower levels of social well-being than participants without a self-reported diagnosis in all four regression models. Likewise, participants with a chronic condition had significantly higher levels of loneliness and lower levels of social well-being than individuals without a chronic condition. In contrast, participants infected with COVID-19 reported better social well-being than those without infection. Additionally, participants without a partner reported significantly higher levels of loneliness and lower levels of social well-being than participants with a partner in all models (Table 3 and Table 4).

### 3.3. Additional Value of Communication App Data

Nonsignificant LR tests suggested that adding total smartphone communication use time did not enhance the fit of loneliness (M1.0 vs. M1), χ^2^ = 52.97, *p* = 0.10, or social well-being (M2.0 vs. M2), χ^2^ = 1968.97, *p* = 0.18. Adding smartphone use times by type did not enhance the fit of loneliness (M3.0 vs. M3), χ^2^ = 75.86, *p* = 0.55, or social well-being (M4.0 vs. M4), χ^2^ = 3658.80, *p* = 0.53.

## 4. Discussion

This study investigated whether smartphone communication app use was associated with self-reported levels of loneliness and social well-being across various ages during the COVID-19 pandemic. We analyzed data from a cross-sectional app-based questionnaire and averaged smartphone communication use times derived from the CORONA HEALTH APP study [62].

The average score of perceived loneliness of 8.65 was comparatively high in this study population. For instance, data from a representative German survey showed an average loneliness score of 3.00 in 2017 and of 5.42 in 2020 during the early phases of the pandemic in a study population of German adults [7,41]. One possible explanation for the higher perceived loneliness found in the current study is that the prolonged period of the COVID-19 pandemic may have affected the levels of loneliness. Another explanation can be related to the study sample, which included a relatively high proportion of individuals with a lifetime diagnosis of mental disorder or a chronic condition. Both of these variables have been found to influence perceptions of loneliness and social well-being [80,81]. No representative German survey has yet determined average smartphone use times. The results of prior studies mostly refer to college students and younger adults only and focused mainly on social media use. For example, a national representative study of young adults (19 to 32 years) from the US showed that social media was used for an average of 60 min per day [47], while the average use time of social media per day in the present study, which included middle-aged and older adults, was much less (30 min per day). A study that was conducted during the COVID-19 pandemic reported that instant messengers and social media apps were the most frequently used apps among Italian adults aged between 18 and 78 years. The participants had an average use time of 3.30 h of social media use per day and reported using social media more frequently since the beginning of the pandemic [82]. Belgian adolescents also reported a higher social media use time during the pandemic than before the pandemic [83]. In light of these findings, the amount of time spent with social media in this study appears comparatively low and requires further investigation to interpret and relate the times spent with social media during the COVID-19 pandemic to other periods of time. Since investigations on social media and the most commonly used social media apps vary between studies and over time, this represents one future challenge.

Moreover, the results of this study showed that the weekly average smartphone communication app use time (total use time) did not add significant explanatory value to the already well-known indicators of loneliness and social well-being, such as partnership status [5,41,84]. Additionally, having a diagnosed mental disorder (such as depression) or a chronic condition was positively associated with loneliness, as in other studies [80,85,86,87]. A current COVID-19 infection was associated with better social well-being. One possible explanation is that people infected with COVID-19 experience a higher level of attention and support from their social environment. However, this hypothesis requires further research and cannot be addressed sufficiently based on the present data including only six cases.

Different than expected (H1), communication app use was significantly associated with loneliness and social well-being only when considering the age of participants. Hence, the results of this study indicate that the relatively broad indicator of use time may not be fully sufficient to detect associations between smartphone communication app usage and loneliness and social well-being. The use times of the different apps in this study may reflect communication with friends or family and also with other contacts—for example, business contacts, as many private smartphones are also used in the work context [88]. As previously discussed, it might be that the frequency of social network site (SNS) use influences perceived loneliness as well as how these sites are used [53,89]. Yang [53] proposed that research on SNSs, such as Instagram, should include the quantity and quality of comments that people receive as a response to their posts. Additionally, Mohr et al. [89] suggested that it could be valuable for mental health research to include the duration and number of incoming and outgoing phone calls and SMS messages and the number of missed calls in addition to the “raw” use time. For example, Min et al. [90] found that longer phone calls were associated with close social contacts such as family or friends, while shorter phone calls were associated with business contacts.

The results of the present study are only partly in line with Hypothesis H2a, suggesting that associations may differ by type of communication app. Contrary to our expectations, the SMS use time was unrelated to loneliness and social well-being. However, in line with prior research [91], we found a positive relationship between phone call use time and social well-being. The age-related differences in associations between total use time and social well-being can be interpreted as in line with the socioemotional selectivity theory (SST) by Carstensen [92]. This theory states that elderly people focus on selected and intense social contacts, whereas young people continuously aim to expand their social network [92]. This could explain why young people feel a greater discrepancy between desired and actual social contacts and thus report higher levels of loneliness and lower levels of social well-being in connection with high use times, while elderly people show greater consistency and satisfaction with only a few high-quality relationships that they stay connected with via smartphone use. Additionally, compared to older people, younger people seem to feel higher social obligations when using smartphone apps, which may have a negative impact on their subjective perceptions of social well-being [45].

The result showing that higher social media use time was associated with lower levels of perceived loneliness in older participants (H2b) undermines these conclusions. Additionally, in line with the SST, a study by Chang et al. [93] showed that elderly people tended to have a small, select list of Facebook “friends” and that they used this social media platform to strengthen their relationships with pre-existing social contacts, while younger people had a larger list of Facebook “friends”. The authors concluded that social media networks of older people, compromising social contacts from “the real world”, contributed to lower levels of loneliness and to higher levels of well-being in comparison to the social media networks of younger people [93]. The majority of previous studies agree that age has a moderating role on the relationship between smartphone use and loneliness and social well-being [45,51,94] and emphasize the potential of smartphones to enhance the well-being of older adults (older than 63 years) [94].

### Strengths and Limitations

One strength of this study is that the study sample included a broad age range from 18 to 78 years and was therefore not restricted to certain groups (e.g., high school or college students), as has been previously criticized [20,45]. Another strength is the inclusion of a relevant set of control variables such as chronic conditions and partnership status. These control variables made it possible to investigate the additional value of novel predictors, such as the total use time, for explaining variation in feelings of loneliness and social well-being. Furthermore, the inclusion of ten different smartphone apps offered the opportunity to compare different types of smartphone communication. Several previous studies included only one specific type of smartphone communication function [45].

This study also has some limitations. One limitation is the possible presence of population bias due to the convenience sample; thus, the results cannot be seen as representative of the German adult population. Participation was voluntary, the majority of participants had a high education status, and age was not equally distributed across the adult age range. Additionally, the exclusion of smartphone operating systems other than Android could be a form of selection bias. Götz et al. [95] found differences in sex and age among smartphone users with different smartphone operating systems; iOS users were more often female and on average older than Android users [95]. However, sex was almost equally distributed in the current study sample, whereas educational level was not.

Another limitation is related to the categorization of smartphone app types. Certain apps have diverse functions and may be used for several purposes. For example, WhatsApp and Telegram can be used for text messaging and for voice or video calls. Therefore, some apps cannot be categorized exclusively. Future studies should try to differentiate the various usage types. Last, although several apps were included in the analysis, they do not represent a complete list of the frequently used communication apps among the German population, and other frequently used apps should be included in future studies.

To overcome these limitations, further research on passive smartphone data and loneliness could pursue a targeted recruitment strategy to include a more diverse and representative sample size to draw general conclusions about the German adult population. Additionally, more detailed research on the associations between different types of smartphone communication functions and loneliness and social well-being is needed. For example, evidence on video calls and loneliness is lacking [61]. During the COVID-19 pandemic, video calls have been of interest, since they enable people to stay connected de-spite physical distancing measures [96].

The present findings focus on cross-sectional associations and do not allow causal interpretation. For example, it remains unclear whether higher phone call use times enhance social well-being or individuals with high social well-being tend to speak to others on the phone more often. Thus, another future direction could be to investigate the average interindividual differences and communication app use over time to differentiate individual patterns of smartphone use and intraindividual changes that may enhance the explanatory value of mental health outcomes [89]. In addition, we cannot rule out possible measurement bias. Further evidence is needed to determine the robustness of an app-based assessment of loneliness and social-well-being and the correspondence with other measurement methodologies such as paper–pencil questionnaires or face-to-face interviews.

Furthermore, it may be of interest to distinguish between different chronic conditions to better understand their impact on loneliness. This becomes particularly important in consideration of the actual COVID-19 pandemic. Current research suggests that older patients with comorbid chronic conditions, such as cardiovascular diseases or diabetes, may be at increased risk of a severe or fatal COVID-19 outcome [97,98,99]. In response to the COVID-19 pandemic, national public health strategies have suggested the isolation of at-risk patients [100]. Thus, these patients may also be at higher risk of having increased levels of perceived loneliness. In line with this, a study by Wong et al. [101] reported that patients with multiple chronic conditions showed increased levels of loneliness and anxiety during the pandemic.

## 5. Conclusions

The ability to rapidly and remotely measure feelings of loneliness and isolation has become increasingly important in the face of the COVID-19 pandemic and the associated social contact restriction measures. The results of this study indicated that passive smartphone communication use time represents only a broad measure of social contact, with limited potential to add value to the detection of subjective feelings of loneliness and social well-being. However, the results indicate that among the different types of communication apps, social media use represents the most promising indicator of feelings of lone-liness and well-being, at least for older adults. The findings of the present study indicate that higher social media use may be an advantage for older adults to overcome loneliness, whereas younger adults may benefit from less use. The direction of the associations cannot be concluded based on the cross-sectional findings. Thus, the impact of smartphone communication apps on strategies aimed to reduce loneliness among older adults should be further analyzed given their potential.

Research on the use of passive smartphone data to monitor mental health is still in progress and needs further validation. Future research should include data that are more highly differentiated (e.g., content-based data, duration and number of incoming and outgoing phone calls) than use time to fully understand the associations between smartphone-assessed communication behavior and loneliness and social well-being. Further studies should include a sample reflecting the population structure and a complete list of frequently used apps to generate representative findings on a population level.

## Figures and Tables

**Table 1:** Self-reported perceived loneliness and social well-being grouped by sociodemographic characteristics (*n* = 364).

Sociodemographic Characteristics	*n* (%)	Loneliness		Social Well-Being	
		*M* [95% *CI*]		*M* [95% *CI*]	
Age group					
18–29 years	65 (17.86%)	9.23	[8.49; 9.97]	54.49	[48.90; 60.07]
30–44 years	146 (40.11%)	8.66	[8.16; 9.17]	57.13	[53.73; 60.54]
45–59 years	110 (30.22%)	8.55	[7.95; 9.15]	55.53	[51.41; 59.65]
60+ years	43 (11.81%)	8.00	[7.20; 8.80]	59.88	[53.57; 66.20]
Sex					
Female	191 (52.47%)	8.89	[8.48; 9.30]	56.76	[53.98; 59.55]
Male	168 (46.15%)	8.43	[7.94; 8.92]	55.90	[52.33; 59.48]
Diverse	5 (1.37%)	7.00	[2.27; 11.73]	66.67	[52.03; 81.30]
Mental disorder					
Yes	159 (43.68%)	9.26	[8.77; 9.74]	50.79	[47.41; 54.16]
No	205 (56.32%)	8.18	[7.77; 8.59]	60.93	[58.16; 63.71]
Chronic disease					
Yes	188 (51.65%)	9.13	[8.68; 9.58]	53.19	[50.17; 56.21]
No	176 (48.45%)	8.14	[7.71; 8.57]	60.04	[56.87; 63.21]
COVID-19 infection					
Yes	6 (1.65%)	7.67	[3.87;11.46]	73.61	[56.64; 90.59]
No	358 (98.35%)	8.65	[8.33; 8.96]	55.92	[53.63; 58.20]
Education					
Low	31 (8.52%)	9.19	[7.99; 10.39]	45.97	[36.44; 55.50]
Moderate	89 (24.45%)	9.02	[8.37; 9.67]	55.81	[51.11; 60.51]
High	244 (67.03%)	8.45	[8.07; 8.83]	58.09	[55.57; 60.62]
Partnership					
Yes	214 (58.79%)	8.22	[7.83; 8.62]	60.48	[57.66; 63.29]
No	150 (41.21%)	9.26	[8.75; 9.77]	50.83	[47.47; 54.19]
Total	364	8.65	[8.34; 8.97]	56.50	[54.30; 58.70]

Notes. *M* = Mean; *CI* = 95% Confidence Interval.

**Table 2:** Proportions of active app use and average use times grouped by type of communication app.

Communication App	*n* (%)	Average Minutes per Week		Average Minutes per Day	
		*M* [95% CI]		*M* [95% CI]	
Social media	230 (63.18%)	245.83	[207.80; 283.86]	30.73	[25.98; 35.48]
Instant messenger	345 (94.78%)	205.64	[178.51; 232.77]	25.71	[22.31; 29.10]
Video call	19 (5.22%)	9.89	[−0.04; 19.83]	1.24	[−0.01; 2.48]
Phone call	127 (34.89%)	22.41	[1.91; 42.92]	2.79	[0.24; 5.36]
SMS	208 (57.14%)	12.78	[4.22; 21.33]	1.60	[0.53; 2.67]
Total use time	364	367.73	[324.35; 411.11]	45.96	[40.54; 51.39]

Notes. *M* = Mean; *CI* = 95% Confidence Interval.

**Table 3:** Results from multiple regression analyses predicting self-reported loneliness and social well-being by smartphone communication app total use time (average of past seven days) of 359 German adults.

Predictors	Loneliness (M1)				Social Well-Being (M2)			
	*B* (SE)		β	*p*	*B* (SE)		β	*p*
Intercept	7.19	(0.64)		<0.001	59.99	(5.49)		<0.001
Total use time	0.21	(0.16)	0.07	0.18	0.19	(0.96)	0.01	0.84
Age	0.23	(0.88)	0.07	0.80	**−19.24**	**(6.76)**	**−0.88**	**0.005**
Age^2^	−0.60	(0.88)	−0.19	0.49	**21.11**	**(6.57)**	**0.96**	**0.001**
Sex								
Male vs. female	−0.02	(0.33)	<−0.01	0.95	−3.46	(2.33)	−0.07	0.14
Mental disorder								
No vs. yes	**0.93**	**(0.32)**	**0.15**	**0.005**	**−9.29**	**(2.34)**	**−0.21**	**<0.001**
Chronic disease								
No vs. yes	**1.03**	**(0.32)**	**0.17**	**0.001**	**−5.35**	**(2.40)**	**−0.12**	**0.02**
COVID-19 infection								
No vs. yes	−1.40	(1.16)	−0.06	0.22	**21.42**	**(7.10)**	**0.13**	**0.003**
Education								
Low vs. moderate	0.41	(0.62)	0.06	0.52	9.47	(5.18)	0.18	0.06
Low vs. high	0.05	(0.58)	0.01	0.93	**10.02**	**(4.91)**	**0.22**	**0.04**
Partnership status								
No vs. yes	**0.95**	**(0.34)**	**0.15**	**0.005**	**−10.99**	**(2.26)**	**−0.25**	**<0.001**
Age × total use time	1.80	(1.07)	0.54	0.09	**−15.79**	**(7.52)**	**−0.66**	**0.04**
Age^2^ × total use time	−1.98	(1.11)	−0.58	0.07	**17.41**	**(7.51)**	**0.71**	**0.02**
*R* ^2^	0.09				0.16			

Notes. *B* = unstandardized beta coefficient, β = standardized beta coefficient, *SE* = standard error; significant results at *p* < 0.05 are highlighted in bold.

**Table 4:** Results from multiple regression analysis predicting self-reported loneliness and social well-being by type of smartphone communication app use time (average of past seven days) of 359 German adults.

Predictors	Loneliness (M3)				Social Well-Being (M4)			
	*B* (SE)		β	*p*	*B* (SE)		β	*p*
Intercept	7.20	(0.66)		<.001	60.54	(5.63)		<0.001
Social media	0.06	(0.13)	0.02	0.63	−0.01	(0.93)	<0.01	0.99
Instant messenger	0.27	(0.18)	0.09	0.15	−0.32	(1.07)	−0.01	0.77
Phone call	0.04	(0.13)	0.01	0.72	**1.24**	**(0.46)**	**0.05**	**0.008**
Text messages	0.18	(0.30)	0.06	0.56	1.50	(1.36)	0.08	0.27
Age	0.19	(0.99)	0.06	0.84	−17.73	(7.04)	**−0.76**	**0.01**
Age^2^	−0.53	(0.98)	−0.17	0.59	19.57	(6.87)	**0.85**	**0.005**
Sex								
Male vs. female	0.01	(0.34)	<0.01	0.97	−3.32	(2.38)	−0.08	0.16
Mental disorder								
No vs. yes	**0.94**	**(0.34)**	**0.15**	**0.005**	**−9.16**	**(2.39)**	**−0.21**	**<0.001**
Chronic disease								
No vs. yes	**1.04**	**(0.33)**	**0.17**	**0.002**	**−5.51**	**(2.27)**	**−0.13**	**0.02**
COVID-19 infection								
No vs. yes	−1.42	(1.16)	−0.06	0.22	**21.41**	**(7.06)**	**0.13**	**0.003**
Education								
Low vs. moderate	0.41	(0.65)	0.06	0.52	9.03	(5.26)	0.18	0.08
Low vs. high	0.03	(0.60)	0.01	0.96	9.38	(5.03)	0.19	0.06
Partnership status								
No vs. yes	**0.92**	**(0.34)**	**0.15**	**0.008**	**−11.13**	**(2.31)**	**−0.25**	**<0.001**
Age × Social Media	1.58	(0.82)	0.52	0.05	−7.91	(5.81)	−0.31	0.17
Age × Instant Messenger	0.41	(1.37)	0.11	0.76	−10.09	(8.14)	−0.34	0.21
Age × SMS	1.39	(2.24)	0.56	0.54	1.35	(9.33)	0.15	0.86
Age^2^ × Social Media	**−1.88**	**(0.84)**	**−0.59**	**0.03**	7.83	(5.73)	0.30	0.17
Age^2^ × Instant Messenger	−0.29	(1.41)	−0.08	0.84	12.26	(8.50)	0.41	0.15
Age^2^ × SMS	−1.23	(1.95)	−0.56	0.53	−0.09	(8.13)	−0.08	0.98
*R* ^2^	0.08				0.15			

Notes. *B* = unstandardized beta-coefficient, β = standardized beta-coefficient, *SE* = standard error; significant results at *p* < 0.05 are highlighted in bold.

## Data Availability

The data presented in this study are available on request from the corresponding author. The data are not publicly available because participants’ informed consent did not cover public deposition of data.
